# Supplementary figures and images for: A comparison of CXR-CAD software to radiologists in identifying COVID-19 in individuals evaluated for Sars CoV-2 infection in Malawi and Zambia
Source: PLOS Digit Health. 2025 Jan 23;4(1):e0000535. doi: 10.1371/journal.pdig.0000535 (PMC11756753; doi:10.1371/journal.pdig.0000535)

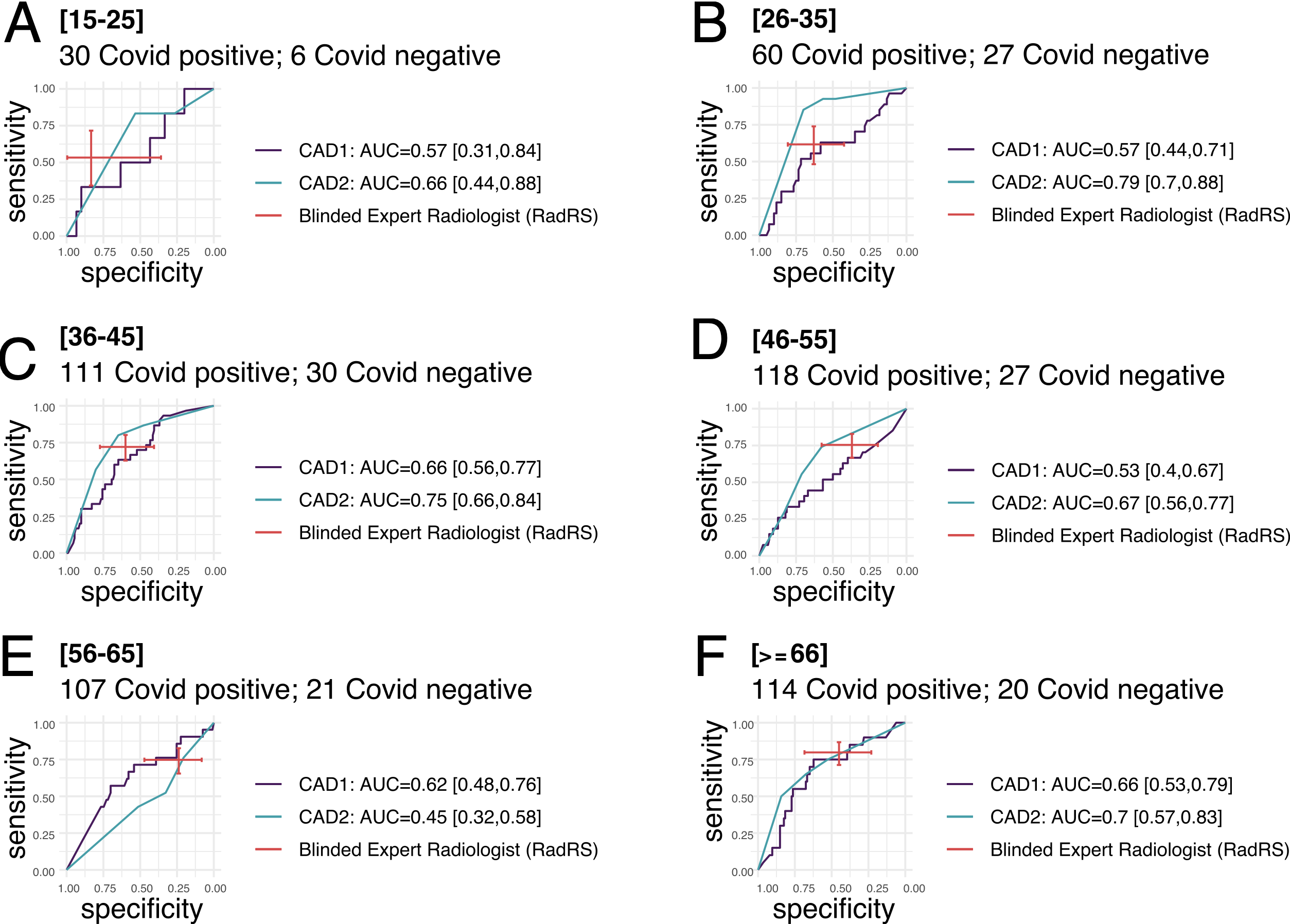

Supplement: S1 Fig — Age 15–25 years (A), Age 26–35 years (B), age 36–45 years (C), age 46–55 years (D) age 56–65 years (E), and age 66 years or higher (F) AUC: Area under Curve, RadRS: Radiologist reference standard for COVID-19. CAD1: Computer Aided Detection software 1, CAD2: Computer Aided Detection software 2. (TIF) [file pdig.0000535.s001.tif]

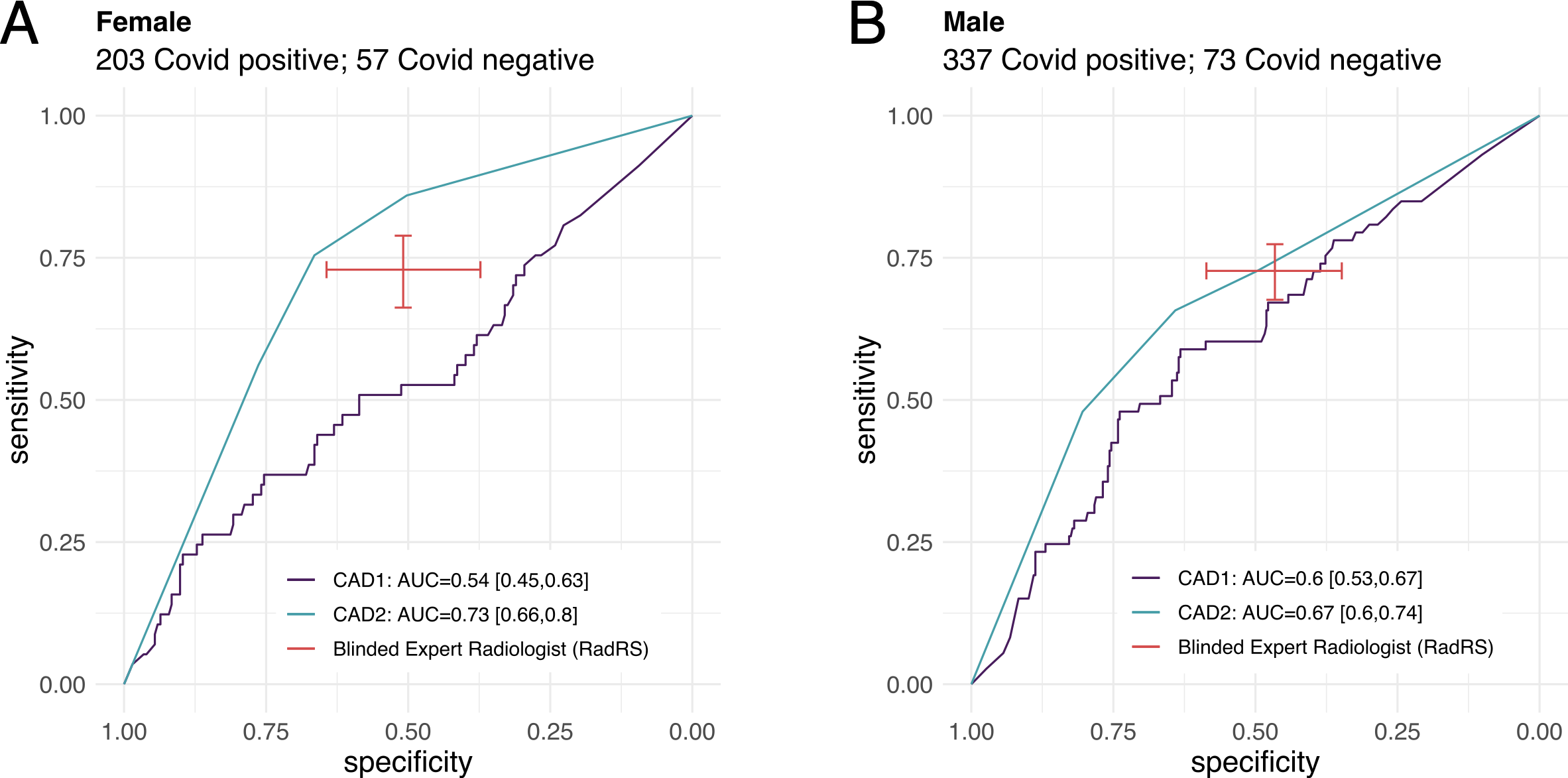

Supplement: S2 Fig — Subgroup analysis of performance of radiologist and software CAD1 and CAD2 at identifying COVID-19 in comparison to baseline molecular testing grouped by gender Female (A) and male (B). AUC: Area under Curve, RadRS: Radiologist reference standard for COVID-19. CAD1: Computer Aided Detection software 1, CAD2: Computer Aided Detection software 2. (TIF) [file pdig.0000535.s002.tif]

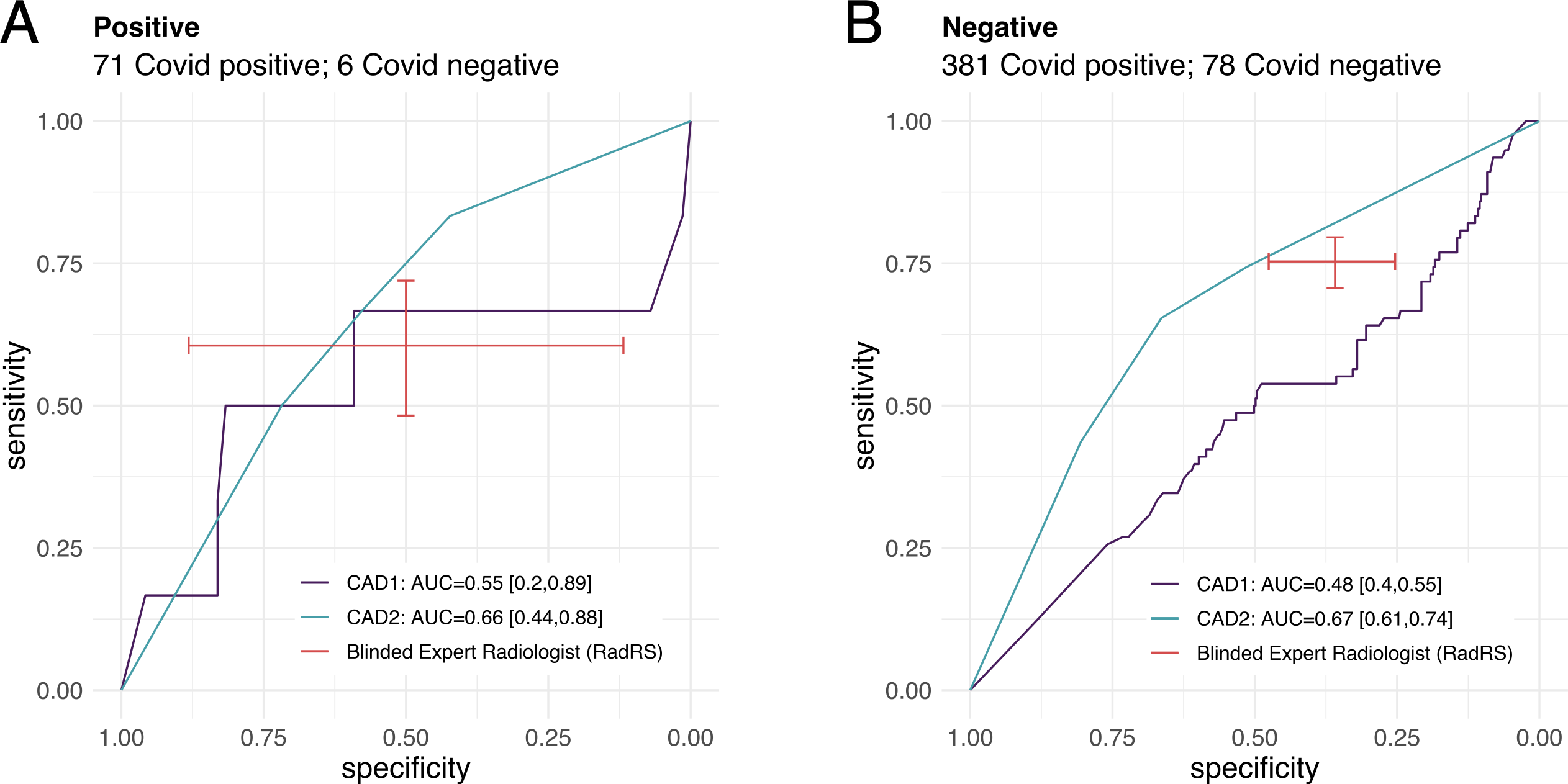

Supplement: S3 Fig — HIV positive (A) and HIV negative (B) AUC: Area under Curve, RadRS: Radiologist reference standard for COVID-19. CAD1: Computer Aided Detection software 1, CAD2: Computer Aided Detection software 2. (TIF) [file pdig.0000535.s003.tif]

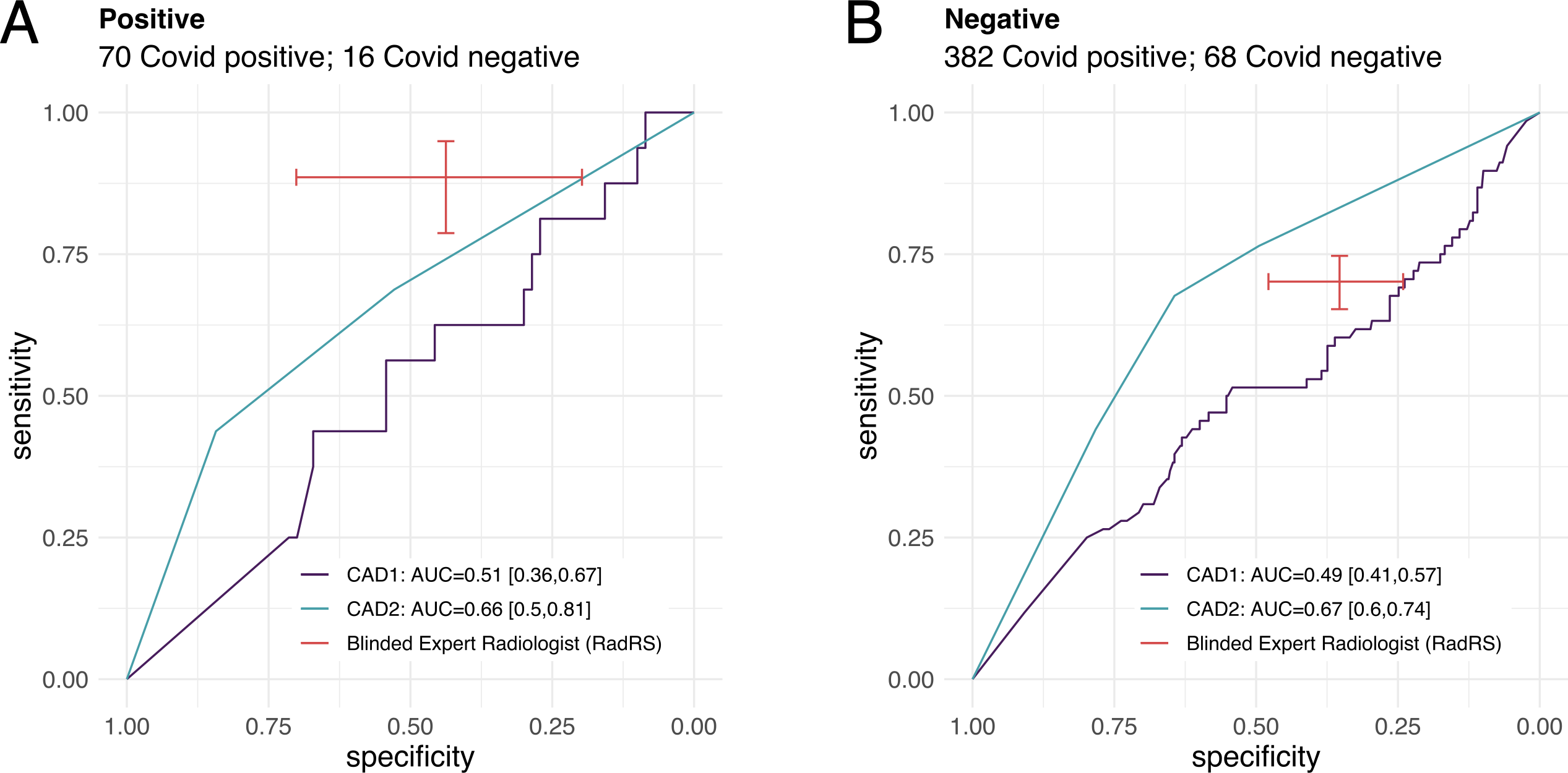

Supplement: S4 Fig — Diabetic (A) and non diabetic (B) AUC: Area under Curve, RadRS: Radiologist reference standard for COVID-19. CAD1: Computer Aided Detection software 1, CAD2: Computer Aided Detection software 2. (TIF) [file pdig.0000535.s004.tif]
