# Supplementary material for: A comparison of CXR-CAD software to radiologists in identifying COVID-19 in individuals evaluated for Sars CoV-2 infection in Malawi and Zambia
Source: PLOS Digit Health. 2025 Jan 23;4(1):e0000535. doi: 10.1371/journal.pdig.0000535 (PMC11756753; doi:10.1371/journal.pdig.0000535)
Supplement: S1 Table — (DOCX) [file pdig.0000535.s005.docx]

|  | **Radiologist assessment** | |
| --- | --- | --- |
|  | **Negative** | **Positive** |
| **CAD1** | | |
| Negative | 95 (45%) | 151 (33%) |
| Positive | 116 (55%) | 309 (67% |
| **CAD2** | | |
| Negative | 162 (77%) | 119 (26%) |
| Positive | 49 (23%) | 341 (74%) |

S1 Table: Agreement of CAD software with radiologists, at manufacturer suggested thresholds for COVID-19 CAD1: Computer Aided Detection software 1, CAD2: Computer Aided Detection software 2
