# Supplementary material for: A comparison of CXR-CAD software to radiologists in identifying COVID-19 in individuals evaluated for Sars CoV-2 infection in Malawi and Zambia
Source: PLOS Digit Health. 2025 Jan 23;4(1):e0000535. doi: 10.1371/journal.pdig.0000535 (PMC11756753; doi:10.1371/journal.pdig.0000535)
Supplement: S2 Table — CAD1: Computer Aided Detection software 1, CAD2: Computer Aided Detection software 2 AC1: Gwet’s Agreement Coefficient. (DOCX) [file pdig.0000535.s006.docx]

| **Software** | **N** | **Agreement** | **AC1** |
| --- | --- | --- | --- |
| **CAD1** | **671** | **60.00%** | **0.28** |
| **CAD2** | **671** | **75.00%** | **0.53** |

S2 Table: Percentage agreement of CAD software with radiologists, at manufacturer suggested thresholds for COVID-19 and the calculated agreement coefficient. CAD1: Computer Aided Detection software 1, CAD2: Computer Aided Detection software 2 AC1: Gwet’s Agreement Coefficient.
