# Supplementary material for: A comparison of CXR-CAD software to radiologists in identifying COVID-19 in individuals evaluated for Sars CoV-2 infection in Malawi and Zambia
Source: PLOS Digit Health. 2025 Jan 23;4(1):e0000535. doi: 10.1371/journal.pdig.0000535 (PMC11756753; doi:10.1371/journal.pdig.0000535)
Supplement: Attachment — Submitted filename: PDIG-D-24-00202_Response to reviewers.docx [file pdig.0000535.s007.docx]

Response to Reviewers

1. Does this manuscript meet PLOS Digital Health’s publication criteria? Is the manuscript technically sound, and do the data support the conclusions? The manuscript must describe methodologically and ethically rigorous research with conclusions that are appropriately drawn based on the data presented.


   Reviewer #1: Partly

   Reviewer #2: Yes

   --------------------
2. Has the statistical analysis been performed appropriately and rigorously

   Reviewer #1: No

   Reviewer #2: Yes

   --------------------
3. Have the authors made all data underlying the findings in their manuscript fully available (please refer to the Data Availability Statement at the start of the manuscript PDF file)?

PLOS Data policy requires authors to make all data underlying the findings described in their manuscript fully available without restriction, with rare exception. The data should be provided as part of the manuscript or its supporting information, or deposited to a public repository. For example, in addition to summary statistics, the data points behind means, medians and variance measures should be available. If there are restrictions on publicly sharing data—e.g. participant privacy or use of data from a third party—those must be specified.

Reviewer #1: Yes

Reviewer #2: Yes

--------------------

4. Is the manuscript presented in an intelligible fashion and written in standard English?

PLOS Digital Health does not copyedit accepted manuscripts, so the language in submitted articles must be clear, correct, and unambiguous. Any typographical or grammatical errors should be corrected at revision, so please note any specific errors here.

Reviewer #1: Yes

Reviewer #2: Yes

--------------------

5. Review Comments to the Author

Please use the space provided to explain your answers to the questions above. You may also include additional comments for the author, including concerns about dual publication, research ethics, or publication ethics. (Please upload your review as an attachment if it exceeds 20,000 characters)

Reviewer #1:

The authors performed a retrospective study to assess the performance of two software products in the interpretation of chest radiographs performed in Zambia and Malawi during the COVID-19 pandemic. The concordance of radiologist and software interpretations of CXR with respect to normality and COVID-19 specifically was assessed, as was the concordance of CXR interpretation with COVID-19 microbiologic testing. To the extent that such software products were and may in the future be used for this purpose, it makes sense to critically appraise their performance. **However, the project of diagnosing COVID-19 via chest x-ray, whether by a human or by a computer, seems fundamentally misguided.** As we’ve learned since 2020, clinically significant disease caused by SARS-CoV-2 can be consistent with a wide array of CXR findings including normal studies. Additionally, PCR positivity for SARS-CoV-2 can be consistent with acute disease, asymptomatic infection, and resolved past infection. Because of this underlying biology, it is difficult to know what to make of concordance or lack of concordance between CXR classification and microbiologic test results. While in the abstract there is a need to understand how radiology interpretation software of this type performs in the clinic, **this study is hampered in that project by the fundamental unsuitability of any method of CXR interpretation to make the diagnosis of COVID-19.**

The retrospective nature of the data collection also makes interpretation challenging. The study population included adults who presented to one of seven sites “who had symptoms consistent with COVID-19 based on a clinical evaluation” and for whom both a CXR and microbiologic test for SARS-CoV-2 was available. However, the symptoms assessed are not clear and Table 1 records that 85 of the included patients had either no symptoms or no record of symptoms. Also unclear is how both CXR and microbiologic testing were deployed. Over the course of the study period, many physicians around the world altered how they assessed COVID-19 in response to changing availability of resources, an evolving understanding of the disease, and alterations in patient presentation due to prior exposures and viral variation. It is clear that the subject population included in this study is heterogeneous. Per Table 1, at three sites 100% of the contributed cases were positive for COVID-19 by
microbiologic testing. At one site, only 17% of contributed cases were positive. This suggests quite different underlying patient populations or, more likely, very different functional inclusion criteria due to practice patterns around symptom assessment and testing. While all included cases were assessed by both software models and radiologists, distinct groups of radiologists were used for cases from Zambia and Malawi. In particular for the analyses stratified by country, these practice pattern or patient population differences would be expected to affect some results of the study.

- *We would like to thank reviewer 1 for their clear feedback and shared concerns regarding the current study. We agree with your comment that there are challenges in applying radiology as a screening tool for COVID-19, given the variable respiratory involvement of the disease, and variability respiratory penetrance by strain. However, we feel there are a number of reasons, despite these limitations, that this study is still of value. First, we would argue that in many instance, chest radiograph remains a first test used in the evaluation of respiratory symptoms, especially in settings with a high burden of TB, including populations in Zambia and Malawi. Furthermore, CXR CAD is recommended as an alternative to a human reader for the screening and triage of adults in high burden TB settings, and many CXR-CAD software have other features (including modules to evaluate COVID risk during the pandemic). As a result, and in the context of COVID-19 modules, it is valuable to ask how such software would perform in these settings in comparison to a radiologist, given the variable access to other diagnostics for respiratory infections globally and the potential for additional uses of CXR CAD in such settings. Second, we would also argue that many respiratory pandemics (including Sars CoV1, Sars CoV2, and MERS-CoV) present with radiographic findings and a better understanding of how CXR and CAD software performed in the wake of the Sars CoV2 pandemic can better inform future respiratory pandemics. In response to these concerns from the reviewer, we have added expanded text addressing these points in the introduction.*

*In response to your comments above, we appreciate that our findings need a careful interpretation and warrant further exploration towards understanding the application of CAD as an in diagnostics related to emerging public health threats. Given the change in Sars CoV2 strains and the variable phenotype impacted by a number of factors, this study is meant to shed light on IF such commercial software that are current in use in many countries globally worked in the African context and IF this could be a viable consideration for non-TB respiratory infections (including respiratory pandemics) in the future. Given the widespread availability of CXR data, the abnormality detection capacity based on CXR-CAD could benefit diagnostic decision making in absence of specific assays, i.e, in the early stage of an emerging pandemic. Given the timing of this data collection, and the evolution of the pandemic, a prospective study would likely not have resulted in adequate number of cases and controls for evaluation. However, we agree with the many limitations in the study design and have added these to the limitations in the document.*

- *We also like to thank the reviewer for pointing out the absence of symptoms among the COVID-19 patients in our study. All patients presented at the clinic due to health complaints and had a presentation that the clinician deemed consistent with COVID-19 and had a provider initiate both COVID-19 testing and a CXR given the patient’s presentation. It is possible that in some who were listed as asymptomatic, or else in which no result was recorded for symptoms, provider findings on clinical exam drove further evaluations. For example, in 18 of the 22 individuals who were Sars CoV-2 positive, but in whom symptoms were absent, the provider ultimately gave these patients a clinical diagnosis of pneumonia. Additionally, in 33 of 39 individuals with positive Sars CoV-2 testing in whom no result was recorded for whether symptoms were present or absent, a clinical diagnosis of pneumonia was given. Therefore, we believe that medical records may have been incomplete in these cases. We have reflected this in the revised manuscript.*
- *Given this was a retrospective study, we focused data collection on patients which met inclusion criteria. As a result, all included patients were deemed by a clinician to have a presentation consistent with COVID-19 and who had both a CXR and COVID-19 test as defined in the section listed as “reference standards”. This is detailed in the ”study design and participants” section of the manuscript. Microbiologic reference standards were defined as WHO approved Sars CoV2 testing (these tests are listed in reference 17).*
- *The concerns regarding patient populations, localized assessments and distinct groups of radiologists resonate with our own impression with the data origins for this study. An underlying concern is matter of continuous debate, i.e. to what extent is the vigorous standardization representative of real-world settings and resources. When this study was conceived, we aimed to collect data retrospectively from multiple sites in a way that would allow us to evaluated for heterogeneity in data. This was, in part, because reported cases of COVID-19 had decreased significantly near the time of study initiation, and a prospective study was not possible which would capture an adequate number of cases and controls within the period of the data collection. Additionally, we shared standard approaches to CXR evaluation with radiologists (see reference 18). While we acknowledge that having a common radiologist pool evaluated all images, we felt that , pragmatically, comparing software to expert radiologists in country was also of value, given the potential role as an alternative tool for use if a radiologist is not available.*

  Several more minor points:
  •       40 cases were excluded from the study because images could not be processed by the software used. This factor seems relevant to software performance and more detail about this limitation would be interested.
- *All of the unprocessed images were intact DICOM files but were not recognized as a valid chest X-ray image from adults by either one of the CAD applications. The majority was flagged as such by CAD1 (40/46) and the overlap was small (2).*

•       The antigen and PCR tests for SARS-CoV-2 have very different performance characteristics as is obvious from Table 1, and concordance of CXR abnormalities with positivity by these two modalities would be expected to differ as well. Stratifying the analysis by type of COVID-19 test would substantially strengthen the manuscript.

- *We would like to thank the reviewer for their observation. Once stratified, the performance of both CAD shows alignment in the subgroup for which Antigen test had been made available as molecular benchmark. Antigen testing seems furthermore to relate to a better performance among radiologist assessments. We have added this information to the results.*

•       Software performance is discussed as being “non inferior” to the radiologist standard. A noninferiority margin should be explicitly defined and defended.

- *We have clarified this wording in the results.*

•       The analysis stratified by time (Figure 4) is discussed in terms of the circulating viral strain being responsible for differences in software performance. But this is not justified. More than the viral strain differed over time during the pandemic, including patient triage practices, test availability, vaccination rates, and rates of prior exposure to the virus.

- *We agree with this point and have added the following text to the discussion section.*
  - *“Coinciding with the time that Omicron superseded the Delta strain as the globally dominant variant, the rate of infections and clinical handling started to show a different dynamics: new strains were more infectious but may have resulted in decreased severity of disease, and immunization through prior exposure rates and vaccinations started to increase, triage practices and molecular test availability became more widespread. The generally milder symptomatic presentation may have resulted in a reduced specificity of radiologists; and a possible increase availability of antigen testing improved the benchmark for diagnosing acute disease and thereby improved the performance of CAD1.”*

•       Several variables would be expected to interact to affect the sensitivity/specificity of COVID-19 diagnosis by CXR including the immune experience of the patient population over time, clinical practice as discussed above, viral strain, and COVID-19 test modality. However, it is never clear how these factors interact for any particular analysis. For example, was the Omicron strain equally represented in cases from Zambia and Malawi? Was PCR testing used with equal frequency across the analysis categories?

- *Although there are no data on strain in our data, at the time of, the WHO first discovered the Omicron strain in November of 2021 in Botswana and was reported as the dominant strain in South Africa by mid November of 2021. Given the transmissibility and the this regional data on predominant strains, we felt use of that use of the November timepoint was a reasonable surrogate for the strain associated with Sars CoV2 testing. We’ve updated the description to clarify this. Additionally, although we agree that other factors may have associated with immune response over time, we felt that our cross over design, where each CXR was evaluated independently by a radiologist and the software and then compared, would partially address variations in clinical presentation over time.*

•       I am unclear on the justification for using distinct radiologist groups as the reference standard for Zambia and Malawi cases. This decreases power for the stratified analysis and introduces another potential bias to the pooled analyses. A common radiologist reference group would surely be preferable.

- *As noted above, we do agree that having a common radiologist had advantages in pooled analysis. However, in the study design, we opted to have in country radiologists involved in interpretation given available resources and data sovereignty laws. Despite this fact, we see there is value in comparison of the software performance to expert radiologists in country to understand how these two measures would compare.*

In sum, I think the authors make a convincing case that the two software products tested performed differently. However, conclusions beyond this are difficult to assess with the analyses presented.

Reviewer #2:

This is a very well written study comparing the performance of different AI-guided tools to diagnose COVID-19 by chest x-ray with an experienced radiologist. The statistical methods were strong and the results were overall clearly communicated. Some important limitations should be addressed before recommending publication.

Description is lacking in terms of the reference diagnostic process. Nearly all of the sample population was diagnosed with COVID-19 by nasal swab or nasopharyngeal swab, which can capture a wide range of presentations due to COVID-19 including asymptomatic disease, mild upper respiratory illness, and lower respiratory tract disease in addition to shedding without the disease. Three of those four categories would have no chest x-ray findings at all. It's helpful that the analysis limited comparisons to the same ground truth. If the data is available, then inclusion of the range of clinical criteria prompted microbiologic testing or a distribution of the population's presenting symptoms would strengthen the foundation of the study significantly to better understand the prior probabilities of lower respiratory tract COVID-19.

- *This is an important point. As shown in table 1, we had limited data on the sampling method used, aside from nasal swab or nasopharyngeal swab. Given our inclusion criteria, we selected individuals evaluated for COVID-19 and in whom clinical providers identified a need for both Sars CoV-2 testing and a chest radiograph. While we acknowledge that this approach is imperfect, we presume that the addition of medical imaging in these patients suggested a clinical concern over lower respiratory disease.*

It's very helpful to note the lack of transparency regarding how these two models were trained. Is information such as the type of model or year that the models were published available? Such as a brief description broadly on whether these were convolutional neural networks, computer vision APIs, or something else.

- We appreciate the reviewer comments and have added language regarding the lack of transparency regarding the developer models. Our understanding is that these models were updated during the course of the pandemic captured in the period of this data collection. However, we have no visibility into how frequently or with what data this software was updated. Generally, our understanding of CAD software used in populations studied at FIND involve Convolutional Neuro Networks, but this may also vary by developer.

Would define the DICOM and FIND abbreviations prior to usage.

- We have defined the DICOM abbreviation. As of 2019, FIND is no longer identified by our previous acronym (the Foundation for Innovative New Diagnostics) and instead uses the stand alone term “FIND”.

Would clarify usage of the term "secondary analysis" which in one location describes it as evaluating the CXR-CAD for agreement with the radiologist and in another location uses the term to describe stratification by strain.

- Thank you for this point, we have changed the language regarding strain to clarify that analysis.

Figure 1 is not immediately intuitive to communicate that participants who were both included and excluded are listed on the same diagram. A flow chart may be more clear, but otherwise a revised figure caption would also be helpful. Something like "the distribution of participants based on available data" would suffice, written prior to defining the categories as you have done.

- Thank you for this point. The figure capture has been updated.

Would be careful of language stating "non inferior" unless a non-inferiority trial is being conducted, in which case a description of the margin for non-inferiority with statistical justification would be required.

- Agreed and thank you also for this point. We have changed the language and removed the “non inferiority” wording from the results.

Where it is written "CAD2, which demonstrated a non-significant trend towards better overall performance, had an observed AUC...," it isn't clear to me which test is being referenced. Is this referring to the overall performance in figure 1 or specifically to figure 4b after listing the results of CAD2 in 4a? If the testing being referenced was a two-hypothesis statistical test, then it can't be certain if there is a trend or not.

- Thank you. We have attempted to clarify this point in the “software interpretation of CXR images for COVID-19:” section.

Otherwise there are several editorial notes that should be addressed. In many locations there are erroneously two spaces between words rather than one, a space between the period and the reference, a space before a period, inconsistent hyphenation of CXR-CAD or CXR CAD, and inconsistent format of the term SARS-CoV-2 and COVID-19. The figure and table captions are inconsistently formatted related to capitalization, spacing, italicization, in addition to the references in the text. The word "includes" in the table 1 caption and in the second line of the "Software interpretation..." section seems like it should say "included" and for figure 4 the abbreviations should be explicitly defined in the caption (they are listed in long form).

- Thank you for this point. We have attempted to address the editorial points in the updated manuscript.

Otherwise the study is innovative, applicable for a highly relevant population, and has rigorous and focused statistical methods with acknowledged limitations. I look forward to the response.

--------------------

6. PLOS authors have the option to publish the peer review history of their article what does this mean?. If published, this will include your full peer review and any attached files.

   Do you want your identity to be public for this peer review?

If you choose “no”, your identity will remain anonymous but your review may still be made public.

For information about this choice, including consent withdrawal, please see our Privacy Policy.
